# Oral Granular Cell Tumour on the Mandibular Gingiva in an Adult: A Rare Case Report

**DOI:** 10.1155/crid/3774958

**Published:** 2025-04-11

**Authors:** Yosuke Iijima, Keisuke Sawada, Mami Yamazaki, Miki Yamada, Shunsuke Hino, Norio Horie, Takahiro Kaneko

**Affiliations:** ^1^Department of Oral and Maxillofacial Surgery, Saitama Medical Center, Saitama Medical University, Saitama, Japan; ^2^Department of Pathology, Saitama Medical Center, Saitama Medical University, Saitama, Japan

**Keywords:** gingiva, granular cell tumour, oral region

## Abstract

**Introduction:** Granular cell tumours are rare benign tumours. In the oral cavity, these tumours are predominantly found on the tongue. This report describes a rare case of a granular cell tumour arising on the gingiva.

**Case Presentation:** A 75-year-old man was referred to the department with a mandibular gingival mass. Intraoral examination revealed a relatively firm mass in the lingual gingiva around the left mandibular premolar. Biopsy results led to a diagnosis of granular cell tumour.

**Conclusion:** Oral granular cell tumours can, in rare cases, occur in areas other than the tongue, such as the gingiva.

## 1. Introduction

Granular cell tumours are a rare benign soft tissue tumour derived from Schwann cells, and the overall incidence of granular cell tumours in surgical specimens has been reported as 0.03% [1, 2]. Histologically, tumour cells contain abundant fine eosinophilic granules in the cytoplasm and are immunobiologically positive for S-100 protein. Recent studies using whole-exome sequencing and targeted sequencing analysis have identified inactivating mutations of ATP6AP1 and ATP6AP2 as likely oncogenic drivers of granular cell tumours [3, 4]. Granular cell tumours are mainly found in the skin (30%–40%), gastrointestinal tract (5%–11%), breast (15%), and respiratory tract (10%), with a predilection for the oral cavity [2, 5]. In the oral cavity, the vast majority of cases arise on the tongue, with a small number of reports describing the involvement of other parts of the oral cavity [6]. We describe a case of granular cell tumour arising in the gingiva, which is relatively rarely reported [7, 8].

## 2. Case Report

A 75-year-old man was referred to the department through his family dentist after a mass noticed on the left mandibular gingiva of the mouth 1 month earlier had not disappeared. The patient had hypertension, was taking amlodipine, and had been smoking 13 cigarettes daily since the age of 20. His alcohol consumption had recently been 350 mL/day of beer.

**Figure 1:**
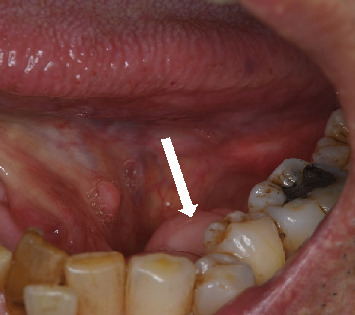
Initial photograph showing a nodular lesion on the lingual gingiva around the left mandibular premolar (arrow).

**Figure 2:**
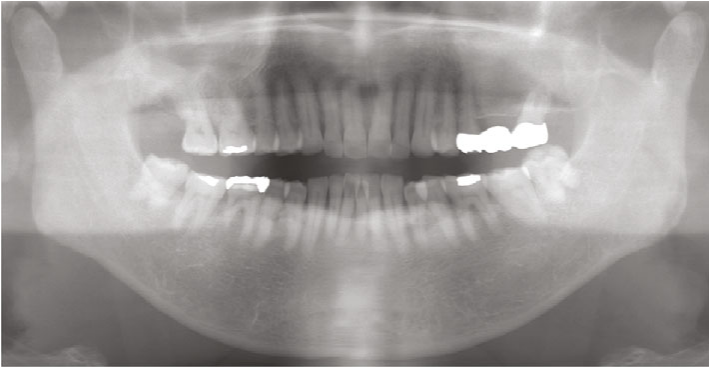
Orthopantomography shows no abnormal bone findings in the region of the left lower first premolar.

Intraoral findings showed a relatively firm mass on the lingual gingiva around the mandibular left premolar, with normal mucosal colouration and a diameter of 10 mm, away from the floor of the mouth (Figure 1). No other abnormalities were evident in the oral region, including in the regional lymph nodes. Radiological examination showed no abnormalities of bone around the left lower first premolar (Figure 2).

**Figure 3:**
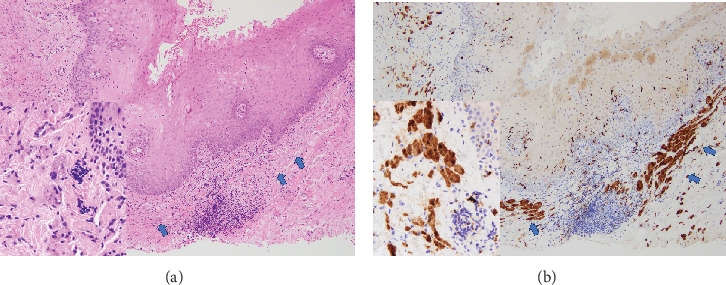
Histopathological staining. (a) Nonencapsulated tumours (arrows) are present under the oral epithelium. The inset shows that tumours comprise nests and sheets of large polygonal cells characterised by uniform nuclei and abundant eosinophilic granular cytoplasm (haematoxylin and eosin stain and original magnification ×100; inset, original magnification ×400). (b) Tumour cells (arrows) are positive for S-100 protein (original magnification ×100; inset, original magnification ×400).

The mass was excised under local anaesthesia with a clinical diagnosis of gingival fibroma. Histopathologically, the excised specimen was nonencapsulated and showed a thin, widespread distribution of polygonal cells with central small dark nuclei and cytoplasmic eosinophilic granules. No evidence of cytological atypia, mitotic activity, or necrosis suggestive of malignant transformation was observed (Figure 3a). Immunohistochemically, tumour cells were positive for S-100 protein (Figure 3b). The histopathological diagnosis was granular cell tumour. Twelve months after removal, no recurrence or malignant transformation was observed.

## 3. Discussion

According to a systematic review by Lafuente-Ibáñez de Mendoza et al., oral granular cell tumours are more common in women (74% female and 26% male), with a mean age at onset of 35.24 years. The predominant site in the oral cavity is the tongue (76%), followed by the lips (11%), the buccal area (5%), the palate (4%), the floor of the mouth (3%), and the gingiva (1%) [6]. Cases involving the gingiva appear rare, with only three reports, including the present [7, 8]. Clinically, granular cell tumours on the tongue were found as asymptomatic, slightly firm, well-delineated submucosal nodules of about 10 mm in diameter. The tumour in the present case was likewise recognised as a nodular lesion on the gingiva, but as very few cases have involved the gingiva, accumulation of more cases is required for valid characterisation. Most cases of granular cell tumour involved solitary lesions, but some showed multiple intra- and extraoral manifestations [9–11]. In addition, multiple granular cell tumours have been shown to be associated with syndromes such as Noonan syndrome, Neurofibromatosis Type 1, and LEOPARD syndrome [2, 12].

Histopathologically, a granular cell tumour presents as an infiltrative, nonencapsulated, submucosal lesion comprising sheets, nests, or trabeculae of large, polygonal cells with abundant, eosinophilic, finely granular cytoplasm [6, 13]. These tumour cells are immunohistochemically positive for S-100 protein (supporting the neural origin of granular cell tumour), as well as for neuron-specific enolase, CD68, calretinin, CD57, inhibin, TFE3, SOX, CD59, PGP9.5, and vimentin [7, 14, 15]. Granular cell tumours of the oral cavity that do not stain for S-100 protein but are positive for CD68 and vimentin have also been reported recently. Some researchers have suggested that the granules are derived from intracytoplasmic lysosomal granules [16]. In one-third of cases of oral granular cell tumours, pseudoepitheliomatous hyperplasia of the covering epithelium is observed. This pseudoepitheliomatous hyperplasia is readily misidentified as squamous cell carcinoma, so care should be taken with the depth of the biopsy [6, 17]. No pseudoepitheliomatous hyperplasia of the covering epithelium was observed in the present case.

The differential diagnosis of granular cell tumour arising in the gingiva requires differentiation from various odontogenic and nonodontogenic mass-forming proliferative diseases. Radiographic examination facilitates diagnosis, but whether this pathology shows specific bone findings remains unclear, as granular cell tumours arising in the gingiva are extremely rare. For example, radiopaque images may help with differentiation from osteogenic lesions such as peripheral ossifying fibroma, while radiolucent images may help with differentiation from malignant tumours, including metastatic ones. In any case, a histopathological search is essential. Aside from diseases within the jawbones, diseases with granular cells in adult soft tissues include primitive non-neural granular cell tumours, adult rhabdomyomas, and malignant melanomas. Primitive non-neural granular cell tumours are negative for S-100 protein [18]. Adult rhabdomyomas are positive for desmin and myogenin [19]. Malignant melanomas with occasional granular cell changes show diffuse positivity for S-100 protein and positivity for melanocytic markers [13].

Granular cell tumours have been reported to have a malignant aspect in less than 2% of all cases, and histological criteria for diagnosis have also been provided. Six histological criteria are assessed in the Fanburg–Smith criteria: necrosis, spindling, vesicular nuclei with large nucleoli, increased mitotic activity (> 2 mitoses/10 high magnification fields at ×200 magnification), high nuclear-to-cytoplasmic (N:C) ratio, and pleomorphism. Neoplasms meeting three or more of these criteria are histologically classified as malignant, while those meeting one or two criteria are classified as atypical, and those showing only focal pleomorphism and not meeting any other criteria are classified as benign [17]. The neoplasm in the present case was judged to be benign. No reports have described malignant cases in the oral cavity, but one case of an aggressive tumour with ulceration of the buccal mucosa has been reported, although it was not histologically classified as malignant [6, 20].

In terms of treatment, complete surgical resection is recommended due to the proliferative nature of the lesion.

In conclusion, oral granular cell tumours can, in rare cases, occur in areas other than the tongue, such as the gingiva.

## Data Availability

The data that support the findings of this study are available from the corresponding author.
